# Mapping the Prevalence and Sociodemographic Characteristics of Women Who Deliver Alone: Evidence From Demographic and Health Surveys From 80 Countries

**DOI:** 10.9745/GHSP-D-15-00261

**Published:** 2016-03-25

**Authors:** Nosakhare Orobaton, Anne Austin, Bolaji Fapohunda, Dele Abegunde, Kizzy Omo

**Affiliations:** a JSI Research & Training Institute, Inc., United States Agency for International Development (USAID) | Targeted States High Impact Project (TSHIP), Washington, DC, USA; b JSI Research & Training Institute, Inc., USAID | TSHIP, Boston, MA, USA; c JSI Research & Training Institute, Inc., USAID | TSHIP, Abuja, Nigeria

## Abstract

Evidence has shown that quality skilled care during labor and delivery is essential to improve maternal and newborn health outcomes. Unfortunately, analyses of Demographic and Health Survey (DHS) data show that there are a substantial number of women around the world that not only do not have access to skilled care but also deliver alone with no one present (NOP). Among the 80 countries with data, we found the practice of delivering with NOP was concentrated in West and Central Africa and parts of East Africa. Across these countries, the prevalence of giving birth with NOP was higher among women who were poor, older, of higher parity, living in rural areas, and uneducated than among their counterparts. As women increased use of antenatal care services, the proportion giving birth with NOP declined. Using census data for each country from the US Census Bureau’s International Database and data on prevalence of delivering with NOP from the DHS among countries with surveys from 2005 onwards (n = 59), we estimated the number of women who gave birth alone in each country, as well as each country’s contribution to the total burden. Our analysis indicates that between 2005 and 2015, an estimated 2.2 million women, who had given birth in the 3 years preceding each country survey, delivered with NOP. Nigeria, alone, accounted for 44% (nearly 1 million) of these deliveries. As countries work on reducing inequalities in access to health care, wealth, education, and family planning, concurrent efforts to change community norms that condone and facilitate the practice of women giving birth alone must also be implemented. Programmatic experience from Sokoto State in northern Nigeria suggests that the practice can be reduced markedly through grassroots community advocacy and education, even in poor and low-resource areas. It is time for leaders to act now to eradicate the practice of giving birth alone—one of many important steps needed to ensure no mother or newborn dies of a preventable death.

## Abstract (precis)

An estimated 2.2 million women surveyed in low- and middle-income countries between 2005 and 2015 gave birth alone. This practice was concentrated in West and Central Africa and parts of East Africa. Women who delivered with no one present were very poor, uneducated, older, and of higher parity. Experience from northern Nigeria suggests the practice can be reduced markedly by mobilizing religious and civil society leaders to improve community awareness about the critical importance of having an attendant present.

## INTRODUCTION

The United Nations Sustainable Development Goals (SDGs), endorsed in September 2015, provide a framework for improving population health outcomes for billions of people globally. The SDGs cover many topics from poverty eradication to climate change and represent a global consensus on an agenda to reduce inequalities.^1^ Of the 17 SDGs, the third one (ensure healthy lives and promote well-being for all at all ages) explicitly pertains to health outcomes. The first target under SDG 3 (target 3.1) calls for a reduction in the global maternal mortality ratio to fewer than 70 per 100,000 live births by 2030. The second target under SDG 3 (target 3.2) aims to reduce newborn mortality to fewer than 12 deaths per 1,000 live births.^2^

Evidence has shown that quality, skilled care during labor and delivery is a required and key intervention to improve maternal and newborn health outcomes.^3^^,^^4^ Unless every mother and newborn has access to such services, preventable maternal and newborn deaths are likely to continue and will jeopardize the attainment of SDG targets 3.1 and 3.2. Too many women and newborns, particularly in countries with weak health systems, social inequalities, and few available services, cannot access or afford high-quality maternity care.^5^

More alarmingly, there is an additional sub-population of women and their newborns, embedded among those who do not have access to quality skilled care, who deliver absolutely alone with “no one present” (NOP). Delivery with NOP has recently become a focus of interest in Nigeria, although it has been neglected in most global policy and practice discussions.^6^^-^^8^ Published work on women giving birth with NOP in other countries and regions of the world is scant. We believe that this is an omission; women who give birth alone are denied the social support of companionship during birth and have no one to act on their behalf as a timely conduit to the health system in the event of maternal or newborn complications. Under such a scenario, this subset of women is likely to contribute disproportionately to the burden of maternal and neonatal mortality. This paper identifies differentials in the prevalence and socioeconomic characteristics of women who delivered with NOP among the 80 countries with available DHS data. This is an important first step in developing interventions to eradicate the practice, and ultimately in achieving SDG targets 3.1 and 3.2.

There is an additional sub-population of women, embedded among those who do not have access to quality skilled care, who deliver absolutely alone.

## DATA AND METHODS

Since 1984, the Demographic and Health Surveys (DHS) have been conducted in at least 85 countries.^9^ DHS data have documented the association between skilled assistance at delivery and lower rates of mortality and morbidity among mothers and their newborns.^10^^,^^11^ In addition to quantifying the prevalence of skilled birth attendance, the DHS also explicitly collects data on women who gave birth with NOP.

For our analysis, we used publicly available data from the DHS program’s STATcompiler database to profile the distribution of delivery with NOP across countries, as well as to identify which sub-populations within countries were most likely to engage in this risky practice.^12^ Data on women giving birth alone were available for 80 countries. The STATcompiler database also enabled us to stratify all live births that occurred with NOP in the 3 years preceding the most recent country DHS survey on several indicators. The variables available were urban/rural residence, wealth quintile, mother’s age, number of antenatal care (ANC) visits, birth order, and mother’s level of education. Although most countries had full data on these stratification variables, some disaggregated data were missing for Botswana, Ecuador, El Salvador, Mexico, Sri Lanka, Sudan, Thailand, and Trinidad and Tobago.

We also sought to estimate each country’s contribution to the total burden of women who gave birth alone among surveyed countries. In doing this, we used the mid-year population of women between the ages of 15–49, as calculated by the US Census Bureau’s International Database, during the same year as each DHS, adjusted for the general fertility rates (as presented in STATcompiler) for the 3 years preceding each survey year. For these analyses, we found census data for the same year as the DHS data for 77 countries. (Census data were missing for Ecuador, Sudan, and Thailand and were excluded from the analysis because they did not have recent DHS surveys conducted after 2004; see below.)^13^

These numbers were used to calculate a rough estimate of the number of women who would have given birth alone, given the prevalence rates of delivery with NOP at the time of the most recent DHS survey after 2004. We excluded 18 countries in the final analyses (besides Ecuador, Sudan, and Thailand mentioned above) as they had no data available after 2004. These countries were Botswana, Brazil, Central African Republic, Chad, Eritrea, Guatemala, Mauritania, Mexico, Morocco, Nicaragua, Paraguay, South Africa, Sri Lanka, Trinidad and Tobago, Turkey, Turkmenistan, Uzbekistan, and Vietnam. This yielded a total of 59 countries with recent data that were used to assess the number of women giving birth alone.

Data from the DHS and the US Census Bureau are both open access and publicly available. Additionally, as standard protocol, each DHS survey received in-country ethical clearance. As both of these data sources are anonymized, we did not seek any additional ethical approval for this work.

## RESULTS

### Estimated Magnitude of Delivery With NOP

For the 59 countries with data since 2005, we estimated there were 2.2 million deliveries with NOP in the 3 years preceding the most recent country survey (Table 1). On a country-by-country basis, the number of women who gave birth alone in 7 countries (Nigeria, India, Niger, Tanzania, Ethiopia, Uganda, and Kenya) made up 78% of the total number of women who gave birth alone. Although the proportion of women who gave birth with NOP was highest in Niger (at 14.5%), the sheer number of women giving birth alone in Nigeria, estimated at almost 1 million in 2013, conferred Nigeria with a problem of greater absolute magnitude. We estimated that Nigeria alone contributed 44% of the total estimated number of women giving birth alone.^9^ It is also noteworthy that although only 0.5% of women in India gave birth alone in 2005, due to its large population, India contributed 6% of the total number of women giving birth alone.

An estimated 2.2 million deliveries with no one present occurred in 59 countries, with Nigeria accounting for 44%.

**TABLE 1:** Estimated Number of Women Who Gave Birth With No One Present (NOP) and Percent Contribution of Each Country to the Total Number of Births With NOP, Selected Countries With DHS Data Between 2005 and 2015^a^

Country & Survey Year	Population of Women Aged 15–49	General Fertility Rate/1,000 Women Aged 15–44^b^	Percentage of Live Births With NOP	Estimated Number of Women Giving Birth With NOP	Estimated Percent Contribution to the Total Number of Births With NOP Among All Surveyed Countries
Nigeria 2013	39,466,768	0.190	13.00%	974,829	44.22%
India 2005	279,621,419	0.101	0.50%	141,209	6.41%
Niger 2012	3,423,589	0.269	14.50%	133,537	6.06%
Tanzania 2011	10,465,797	0.188	6.70%	131,827	5.98%
Ethiopia 2011	20,405,177	0.161	3.80%	124,839	5.66%
Uganda 2011	7,234,128	0.217	6.80%	106,747	4.84%
Kenya 2008	9,361,636	0.161	6.50%	97,970	4.44%
Angola 2006	3,385,034	0.198	8.40%	56,300	2.55%
Mali 2012	3,524,985	0.214	5.80%	43,752	1.98%
DRC 2013	18,043,728	0.225	0.80%	32,479	1.47%
Guinea 2012	2,518,996	0.176	7.10%	31,477	1.43%
Rwanda 2010	2,650,841	0.151	7.30%	29,220	1.33%
Cameroon 2011	4,993,439	0.180	3.10%	27,863	1.26%
Nepal 2011	7,986,822	0.096	2.90%	22,235	1.01%
Côte d'Ivoire 2011	5,293,915	0.174	2.40%	22,107	1.00%
Ghana 2008	5,654,518	0.136	2.70%	20,763	0.94%
Zambia 2013	3,274,651	0.184	3.10%	18,679	0.85%
Malawi 2010	3,388,791	0.202	2.70%	18,482	0.84%
Yemen 2013	6,040,827	0.206	1.40%	17,422	0.79%
Bangladesh 2011	43,392,926	0.092	0.40%	15,969	0.72%
Burundi 2010	2,068,122	0.203	3.60%	15,114	0.69%
Senegal 2014	3,435,961	0.172	2.50%	14,775	0.67%
Pakistan 2012	48,212,804	0.131	0.20%	12,632	0.57%
Mozambique 2011	5,453,352	0.206	1.10%	12,357	0.56%
Zimbabwe 2010	2,894,645	0.150	2.80%	12,158	0.55%
Indonesia 2012	65,894,656	0.088	0.20%	11,597	0.53%
Burkina Faso 2010	3,688,866	0.206	1.40%	10,639	0.48%
Togo 2013	1,755,425	0.163	3.10%	8,870	0.40%
Madagascar 2008	4,668,384	0.168	0.80%	6,274	0.28%
Haiti 2012	2,576,070	0.117	1.90%	5,727	0.26%
Benin 2011	2,144,241	0.175	1.10%	4,128	0.19%
Egypt 2014	22,030,793	0.127	0.10%	2,798	0.13%
Bolivia 2008	2,478,335	0.121	0.90%	2,699	0.12%
Philippines 2013	24,814,911	0.101	0.10%	2,506	0.11%
Peru 2012	8,124,085	0.086	0.30%	2,096	0.10%
Swaziland 2006	320,632	0.137	4.40%	1,933	0.09%
Colombia 2010	12,024,552	0.074	0.20%	1,780	0.08%
Honduras 2011	2,084,188	0.107	0.60%	1,338	0.06%
Timor-Leste 2009	242,026	0.175	3.00%	1,271	0.06%
Congo 2011	1,084,812	0.182	0.50%	987	0.04%
Gambia 2013	486,629	0.085	1.90%	786	0.04%
Namibia 2013	583,375	0.125	0.90%	656	0.03%
Lesotho 2009	523,654	0.119	1.00%	623	0.03%
Azerbaijan 2006	2,637,985	0.066	0.30%	522	0.02%
Sierra Leone 2013	1,391,263	0.169	0.20%	470	0.02%
Jordan 2012	1,722,911	0.112	0.20%	386	0.02%
Liberia 2013	928,619	0.168	0.20%	312	0.01%
Tajikistan 2012	2,128,742	0.134	0.10%	285	0.01%
Dominican Rep. 2013	2,629,898	0.089	0.10%	234	0.01%
Comoros 2012	181,215	0.142	0.90%	232	0.01%
Gabon 2012	376,360	0.144	0.40%	217	0.01%
Kyrgyzstan 2013	1,487,207	0.125	0.10%	186	0.01%
Moldova 2005	1,083,166	0.055	0.20%	119	0.01%
Guyana 2009	185,875	0.094	0.60%	105	<0.01%
Albania 2008	798,442	0.046	0.10%	37	<0.01%
Armenia 2010	849,511	0.061	0.00%	0	<0.01%
Cambodia 2010	4,088,903	0.105	0.00%	0	<0.01%
Sao Tome and Principe 2008	39,078	0.164	0.00%	0	<0.01%
Ukraine 2007	12,201,772	0.039	0.00%	0	<0.01%
**Total**				**2,204,554**	

a We excluded the following 18 countries because they did not have DHS data since 2005: Botswana, Brazil, Central African Republic, Chad, Eritrea, Guatemala, Mauritania, Mexico, Morocco, Nicaragua, Paraguay, South Africa, Sri Lanka, Trinidad and Tobago, Turkey, Turkmenistan, Uzbekistan, and Vietnam. We also excluded Ecuador 1987, Sudan 1989, and Thailand 1987 both because they did not have recent DHS data and because census data were missing for these countries.

b Number of live births in the 3 years preceding each survey year per woman aged 15–44.

Source of data: Population data from the US Census Bureau; general fertility rate and percentage of live births with NOP from STATcompiler.

### Regional Variations

**FIGURE 1.**
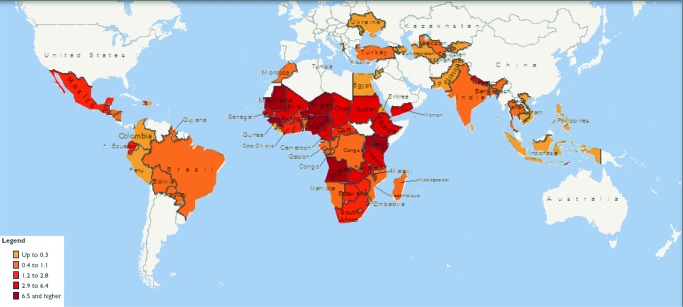
Prevalence of Women Giving Birth With No One Present Among the 80 Countries With Available DHS Data

In Latin America and the Caribbean, the prevalence of women who gave birth alone was less than 3% in each country with survey data, with the exception of El Salvador where it was estimated at 7.6% in 1985 (Table 2). In South and Southeast Asia, Timor-Leste (3% in 2009) and Nepal (2.9% in 2011) had the highest prevalence of women who gave birth alone. Central Asia had a very low prevalence of delivery with NOP, with all countries reporting a prevalence of less than 0.5%, as did most North African, East European, and West Asian countries. Yemen was the main outlier in West Asia, reported at 4.4% of women who gave birth alone in 1997. In general, the highest prevalence of delivery with NOP was found in sub-Saharan Africa, where in 10 of 41 countries with data the prevalence was 5% or higher. The highest levels were in Nigeria at 13% and Niger at 14.5% (Table 2). A mapping of available data that included the 80 countries revealed that delivery with NOP was concentrated in West and Central Africa and parts of East Africa (Figure 1).

**TABLE 2:** Percentage of Women Whose Most Recent Birth^a^ Occurred With No One Present, Stratified by Residence, Wealth Quintile, and Maternal Age

		Residence		Household Wealth Index					Maternal Age at Birth		
Country & Survey Year	Total	Urban	Rural	Lowest	Second	Middle	Fourth	Highest	<20	20–34	≥35
**sub-Saharan Africa**											
Angola 2006-07	8.4	3.5	13.7	15.5	13.0	5.8	3.9	0.6	5.8	9.2	17.3
Benin 2011-12	1.1	0.9	1.3	2.6	1.3	1.0	0.5	0.2	0.7	1.2	1.3
Botswana 1988	1.4	0.2	1.7	NA	NA	NA	NA	NA	0.0	1.6	2.5
Burkina Faso 2010	1.4	0.5	1.5	1.8	2.0	1.2	1.2	0.3	0.3	1.4	1.8
Burundi 2010	3.6	1.6	3.8	5.4	3.7	3.0	4.6	1.3	0.5	3.0	7.4
Cameroon 2011	3.1	1.3	4.4	8.1	3.6	1.6	0.6	0.3	2.2	3.1	4.3
Central African Republic 1994-95	1.8	1.4	2.1	1.5	2.7	2.7	1.4	0.7	0.2	1.9	4.2
Chad 2004	4.0	2.9	4.3	3.6	6.6	4.0	2.7	3.0	2.1	4.5	5.2
Comoros	0.9	0.8	0.9	2.2	0.8	0.2	0.5	0.2	0.0	1.0	1.2
Congo (Brazzaville) 2011-12	0.5	0.1	1.1	1.5	0.4	0.1	0.2	0.0	0.3	0.5	0.7
Congo Democratic Republic 2013-14	0.8	0.5	1.0	1.4	0.9	1.0	0.4	0.3	0.3	0.8	1.4
Côte d'Ivoire 2011-12	2.4	0.7	3.4	3.5	2.6	2.6	2.0	0.6	0.7	2.6	3.4
Eritrea 2002	0.3	0.2	0.3	0.2	0.3	0.4	0.3	0.0	0.5	0.1	0.5
Ethiopia 2011	3.8	1.6	4.2	3.4	4.8	3.8	3.9	2.9	2.8	3.6	6.0
Gabon 2012	0.4	0.3	0.8	0.9	0.2	0.3	0.0	0.5	0.2	0.5	0.3
Gambia 2013	1.9	1.9	1.9	1.9	2.1	2.8	1.7	0.9	0.4	2.0	2.7
Ghana 2008	2.7	1.1	3.8	4.8	3.7	1.7	1.6	0.5	1.1	2.3	5.7
Guinea 2012	7.1	1.9	9.0	11.5	6.5	9.1	5.8	0.5	3.8	6.2	15.8
Kenya 2008-09	6.5	1.8	7.6	8.2	8.4	7.4	5.8	2.1	2.4	5.8	16.4
Lesotho 2009	1.0	0.6	1.1	1.4	0.7	2.0	0.5	0.3	0.5	1.2	0.7
Liberia 2013	0.2	0.0	0.4	0.6	0.1	0.1	0.0	0.1	0.1	0.2	0.3
Madagascar 2008-09	0.8	0.2	0.8	1.7	0.6	0.4	0.5	0.1	0.2	0.7	2.0
Malawi 2010	2.7	2.2	2.8	3.9	3.1	2.4	2.4	1.5	0.5	2.5	6.7
Mali 2012-13	5.8	2.0	6.7	7.7	6.7	8.0	4.5	1.1	3.9	5.8	8.1
Mauritania 2000-01	6.9	1.6	10.9	7.4	10.4	10.0	4.8	1.1	6.6	6.6	8.4
Mozambique 2011	1.1	1.2	1.1	0.5	1.4	0.9	1.2	1.8	0.9	0.9	2.6
Namibia 2013	0.9	0.2	1.5	2.3	0.8	0.4	0.5	0.0	0.0	0.9	1.6
Niger 2012	14.5	3.6	16.2	16.4	17.7	18.1	12.6	7.3	8.8	15.0	19.1
Nigeria	13.0	6.4	16.7	25.7	17.4	10.5	5.1	1.6	9.1	12.8	17.8
Rwanda 2013	7.3	5.2	7.6	9.8	8.9	7.7	5.5	3.1	2.0	6.3	13.7
Sao Tome and Principe 2008-09	0.0	0.0	0.0	0.0	0.0	0.0	0.0	0.0	0.0	0.0	0.0
Senegal 2012-13	5.1	1.5	7.0	11.3	6.7	2.9	2.1	0.2	2.4	4.7	8.7
Sierra Leone 2013	0.2	0.1	0.2	0.3	0.2	0.1	0.2	0.0	0.0	0.2	0.2
South Africa 1998	2.1	0.6	3.5	5.4	2.3	0.5	0.5	0.0	1.1	2.1	3.0
Sudan 1989-90	4.3	2.1	5.5	NA	NA	NA	NA	NA	7.5	3.7	4.5
Swaziland 2006-07	4.4	1.5	5.3	11.3	4.8	1.8	2.1	1.2	2.1	3.7	14.2
Tanzania 2010	3.5	1.1	4.1	4.3	4.5	3.9	3.2	0.0	1.3	3.4	5.5
Togo 2013-14	3.1	0.8	4.4	5.2	5.1	3.4	1.0	0.3	1.5	2.8	5.4
Uganda 2011	6.8	1.0	7.7	8.8	8.7	9.0	5.2	1.1	1.4	6.5	14.2
Zambia	3.1	1.1	4.1	4.5	4.6	3.2	1.2	0.4	0.4	2.5	9.4
Zimbabwe 2013-14	2.8	1.4	3.4	5.1	3.1	3.0	1.2	1.3	1.2	2.5	8.3
**North Africa/West Asia and Eastern Europe**											
Albania 2008-09	0.1	0.2	0.0	0.3	0.0	0.0	0.0	0.0	0.9	0.0	0.0
Armenia 2010	0.0	0.0	0.0	0.0	0.0	0.0	0.0	0.0	0.0	0.0	0.0
Azerbaijan 2006	0.3	0.5	0.1	0.0	0.3	1.2	0.0	0.0	0.0	0.4	0.0
Egypt 2014	0.1	0.0	0.1	0.1	0.3	0.0	0.0	0.0	0.2	0.1	0.2
Jordan 2012	0.2	0.2	0.1	0.2	0.0	0.0	0.7	0.0	0.0	0.2	0.2
Moldova 2005	0.2	0.2	0.2	0.4	0.6	0.0	0.0	0.0	0.0	0.2	0.0
Morocco 2003-04	1.1	0.4	1.8	2.5	1.4	0.4	0.3	0.3	0.0	0.7	3.2
Turkey 1998	0.7	0.3	1.4	2.1	0.7	0.0	0.4	0.0	0.0	0.6	3.3
Ukraine 2007	0.0	0.0	0.0	0.0	0.0	0.0	0.0	0.0	0.0	0.0	0.0
Yemen 1997	4.4	2.7	4.9	3.6	4.1	5.8	5.2	3.5	2.0	4.2	7.7
**Central Asia**											
Kyrgyz Republic 2012	0.1	0.0	0.2	0.0	0.0	0.6	0.0	0.0	0.0	0.2	0.0
Tajikistan 2012	0.1	0.1	0.1	0.5	0.0	0.1	0.0	0.0	0.0	0.1	0.8
Turkmenistan 2000	0.2	0.2	0.1	0.3	0.0	0.0	0.0	0.6	0.0	0.2	0.0
Uzbekistan 1996	0.4	0.0	0.6	0.8	0.0	1.0	0.0	0.0	0.0	0.4	1.9
**South and Southeast Asia**											
Bangladesh 2011	0.4	0.2	0.5	0.7	0.9	0.4	0.1	0.0	0.2	0.6	0.0
Cambodia 2010	0.0	0.0	0.0	0.0	0.0	0.0	0.1	0.0	0.0	0.0	0.0
India 2005-06	0.5	0.3	0.6	1.1	0.5	0.4	0.1	0.1	0.2	0.5	1.4
Indonesia 2012	0.2	0.1	0.4	0.8	0.2	0.1	0.0	0.0	0.4	0.2	0.4
Nepal 2011	2.9	1.4	3.0	7.7	2.6	1.0	0.8	0.3	0.8	3.0	7.2
Pakistan 2012-13	0.2	0.0	0.2	0.1	0.5	0.2	0.0	0.0	0.0	0.2	0.2
Philippines 2013	0.1	0.0	0.2	0.4	0.0	0.0	0.0	0.0	0.0	0.1	0.1
Sri Lanka 1987	0.2	0.0	0.3	NA	NA	NA	NA	NA	0.0	0.3	0.0
Thailand 1987	1.0	0.2	1.1	NA	NA	NA	NA	NA	0.4	0.6	5.0
Timor-Leste 2009-10	3.0	1.6	3.4	4.5	3.5	3.7	2.4	0.6	1.8	2.2	5.6
Vietnam 2002	0.1	0.0	0.1	0.2	0.2	0.0	0.0	0.0	0.0	0.1	0.0
**Latin America and the Caribbean**											
Bolivia 2008	0.9	0.4	1.5	2.0	0.9	0.7	0.0	0.1	0.6	0.9	1.5
Brazil 1996	0.6	0.4	1.0	1.0	0.9	0.3	0.0	0.0	0.2	0.6	0.8
Colombia 2010	0.2	0.1	0.4	0.6	0.2	0.0	0.0	0.0	0.0	0.3	0.2
Dominican Republic 2013	0.1	0.1	0.0	0.1	0.1	0.2	0.0	0.0	0.0	0.1	0.0
Ecuador 1987	2.2	0.6	3.8	NA	NA	NA	NA	NA	0.4	2.0	5.7
El Salvador 1985	7.6	3.0	11.3	NA	NA	NA	NA	NA	4.5	7.8	13.5
Guatemala 1998-99	1.2	0.0	2.0	3.6	1.3	0.3	0.0	0.0	1.0	1.0	2.7
Guyana 2009	0.6	0.3	0.7	0.8	0.0	1.3	0.6	0.0	0.0	0.6	1.8
Haiti 2012	1.9	1.4	2.1	2.3	2.0	1.5	1.4	2.3	0.8	1.5	4.2
Honduras 2011-12	0.6	0.3	1.0	1.5	0.8	0.5	0.0	0.0	0.2	0.7	1.0
Mexico 1987	2.6	0.8	5.6	NA	NA	NA	NA	NA	0.5	2.7	4.9
Nicaragua 2001	1.1	0.5	1.7	2.6	1.2	0.4	0.3	0.1	0.3	1.0	3.8
Paraguay 1990	0.5	0.2	0.8	0.4	1.5	0.0	0.6	0.0	0.0	0.6	0.5
Peru 2012	0.3	0.2	0.7	1.0	0.3	0.0	0.2	0.0	0.0	0.3	0.8
Trinidad and Tobago 1987	0.1	0.0	0.2	NA	NA	NA	NA	NA	0.0	0.1	0.0

a Data were restricted to the most recent live birth in the 3 years preceding each survey.

### Sociodemographic Correlates

Table 2 shows that delivery with NOP is overwhelmingly a rural phenomenon in all the countries with available data. Additionally, we observed a clear association between wealth and delivery with NOP, with the poorest bearing the brunt of the burden in all countries with data. Finally, we found a clear age gradient; the prevalence of delivery with NOP rose as mothers got older in all countries. Furthermore, in 8 sub-Saharan African countries (and El Salvador), the prevalence of delivery with NOP among women over the age of 35 years ranged from 10% to 20%.

Prevalence of delivering alone was higher among women who were poor, uneducated, and living in rural areas than among their counterparts.

When delivery with NOP was disaggregated by residence, wealth, and maternal age, Nigeria emerged as an outlier in terms of the large magnitude of the difference between urban and rural populations that gave birth alone. Nigeria had the highest percentage of both urban (6.4%) and rural women (17%) who gave birth alone. Nigeria also had the largest disparity between wealth categories, wherein 26% of women in the poorest quintile gave birth alone in 2013 in contrast to 2% in the wealthiest quintile. Furthermore, although there was a discernible linear trend showing that as mothers aged, they were more likely to have given birth alone, in Nigeria, as well as in Niger, nearly 10% of mothers under the age of 20 also reported they gave birth alone.

Table 3 presents the distribution of women who gave birth with NOP by number of ANC visits, the birth order of the index child, and maternal education. Across all countries, we observed that as women increased use of ANC, the proportion of women that gave birth with NOP declined. This is most clearly evident in Rwanda, where 36% of the women who reported they had given birth with NOP had not accessed any ANC compared with 9% of women who had made 1–3 ANC visits and 4% of women with 4 or more ANC visits (Table 3). Of equal importance are those factors that enabled women to seek ANC as well as those that removed barriers to accessing ANC. We found that in all countries, women with higher-order births were more likely to have given birth alone. Improvements in mothers’ level of education were associated with reductions in the prevalence of women who gave birth alone across the 80 countries studied.

As women increased use of ANC, the proportion of women giving birth alone declined.

**TABLE 3:** Percentage of Women Whose Most Recent Birth^a^ Occurred With No One Present, Stratified by Antenatal Care (ANC) Visits, Birth Order of the Index Child, and Maternal Educational Levels

		No. of ANC Visits for Recent Birth			Birth Order of the Index Child				Mother’s Highest Educational Level		
Country & Survey Year	Total	None	1–3	≥4	1	2–3	4–5	≥6	None	Primary	Secondary or higher
**sub-Saharan Africa**											
Angola 2006-07	8.4	NA	NA	NA	NA	NA	NA	NA	13.3	7.9	0.0
Benin 2011-12	1.1	4.2	2.0	0.2	0.9	0.9	1.4	1.5	1.5	0.4	0.1
Botswana 1988	1.4	NA	NA	NA	0.3	1.7	1.8	1.9	4.0	0.4	0.0
Burkina Faso 2010	1.4	4.3	1.6	0.5	0.0	1.4	1.7	1.8	1.6	0.3	0.1
Burundi 2010	3.6	16.7	4.0	2.5	0.8	2.3	5.6	6.5	4.6	2.9	0.3
Cameroon 2011	3.1	9.6	3.1	1.5	0.6	2.0	3.2	7.6	8.1	2.0	0.4
Central African Republic 1994-95	1.8	3.7	1.3	1.2	0.2	1.7	2.4	2.9	2.4	1.2	1.0
Chad 2004	4.0	5.1	2.8	2.7	0.9	3.7	4.1	6.3	3.8	5.5	1.4
Comoros	0.9	5.6	0.5	0.3	0.2	0.8	1.1	1.6	1.6	0.6	0.1
Congo (Brazzaville) 2011-12	0.5	3.4	0.6	0.2	0.1	0.4	0.6	1.3	1.7	0.8	0.2
Congo Democratic Republic 2013-14	0.8	2.0	0.6	0.8	0.2	0.4	1.3	1.3	1.2	1.1	0.4
Côte d'Ivoire 2011-12	2.4	8.7	2.7	0.9	0.5	1.8	3.3	4.8	3.0	1.8	0.5
Eritrea 2002	0.3	0.5	0.1	0.2	0.0	0.2	0.0	0.8	0.3	0.3	0.0
Ethiopia 2011	3.8	4.5	2.6	3.4	2.1	3.5	3.1	6.0	4.2	3.3	1.3
Gabon 2012	0.4	2.4	0.3	0.3	0.3	0.2	0.6	0.6	0.2	0.7	0.3
Gambia 2013	1.9	23.9	2.7	1.4	0.6	1.6	1.6	4.1	2.1	1.5	1.9
Ghana 2008	2.7	8.6	4.4	2.1	0.4	0.9	5.8	6.5	4.3	2.8	1.5
Guinea 2012	7.1	20.5	5.6	4.9	2.7	4.9	9.5	12.1	8.7	2.4	1.1
Kenya 2008-09	6.5	17.2	6.7	4.5	0.5	3.7	9.4	16.2	8.0	7.6	3.0
Lesotho 2009	1.0	3.5	1.1	0.6	0.4	0.7	2.7	2.4	3.1	1.3	0.5
Liberia 2013	0.2	1.0	0.6	0.1	0.0	0.1	0.4	0.4	0.2	0.3	0.0
Madagascar 2008-09	0.8	3.6	0.6	0.3	0.0	0.6	0.6	2.1	1.0	0.9	0.2
Malawi 2010	2.7	15.1	2.7	2.3	0.6	1.6	3.2	6.4	4.2	2.7	1.2
Mali 2012-13	5.8	11.8	4.7	3.0	3.6	4.5	6.1	8.9	6.3	4.8	1.5
Mauritania 2000-01	6.9	11.0	5.6	3.0	4.4	7.2	6.3	9.1	9.2	3.3	1.5
Mozambique 2011	1.1	0.4	1.2	1.3	0.7	0.8	1.3	1.9	1.2	1.1	1.1
Namibia 2013	0.9	5.9	2.1	0.5	0.1	0.8	1.8	2.4	4.0	1.9	0.3
Niger 2012	14.5	15.0	15.0	13.7	6.4	11.2	16.7	19.1	15.7	10.1	3.6
Nigeria	13.0	21.5	17.1	6.8	4.3	9.9	14.6	22.6	21.7	9.7	2.6
Rwanda 2013	7.3	36.4	8.7	3.7	1.0	6.2	10.1	15.3	11.7	6.7	2.9
Sao Tome and Principe 2008-09	0.0	0.0	0.0	0.0	0.0	0.0	0.0	0.0	0.0	0.0	0.0
Senegal 2012-13	5.1	23.6	5.8	2.7	1.4	3.2	7.5	9.5	6.3	3.5	0.8
Sierra Leone 2013	0.2	0.0	0.4	0.1	0.0	0.1	0.2	0.3	0.2	0.0	0.0
South Africa 1998	2.1	6.5	2.3	1.7	0.4	1.5	3.5	7.8	7.6	3.1	0.9
Sudan 1989-90	4.3	NA	NA	NA	4.1	4.2	3.8	4.8	7.4	0.4	0.5
Swaziland 2006-07	4.4	15.2	4.6	3.8	0.9	3.1	8.3	12.3	10.6	6.1	2.4
Tanzania 2010	3.5	6.5	4.2	2.2	0.2	2.6	3.6	7.9	3.7	3.7	0.7
Togo 2013-14	3.1	13.5	3.4	1.5	0.5	2.7	2.6	8.4	5.0	2.4	0.9
Uganda 2011	6.8	14.1	8.6	4.3	1.2	3.3	7.4	13.7	12.5	7.2	2.2
Zambia	3.1	7.0	3.5	2.6	0.1	1.1	3.0	9.2	8.0	3.5	0.9
Zimbabwe 2013-14	2.8	5.0	2.9	2.3	0.5	2.0	6.2	10.4	10.7	4.2	2.1
**North Africa/West Asia and Eastern Europe**											
Albania 2008-09	0.1	4.1	0.0	0.0	0.2	0.0	0.0	0.0	6.1	0.0	0.0
Armenia 2010	0.0	0.0	0.0	0.0	0.0	0.0	0.0	0.0	0.0	0.0	0.0
Azerbaijan 2006	0.3	1.6	0.0	0.0	0.0	0.7	0.0	0.0	0.0	0.0	0.3
Egypt 2014	0.1	0.7	0.1	0.0	0.0	0.1	0.2	0.2	0.2	0.1	0.1
Jordan 2012	0.2	1.3	2.3	0.0	0.0	0.3	0.1	0.1	0.0	0.1	0.2
Moldova 2005	0.2	0.0	0.0	0.2	0.0	0.4	0.0	0.0	0.0	0.0	0.2
Morocco 2003-04	1.1	1.8	0.7	0.8	0.0	0.4	1.9	4.6	1.7	0.0	0.1
Turkey 1998	0.7	1.8	0.8	0.0	0.1	0.5	0.7	4.0	1.5	0.7	0.0
Ukraine 2007	0.0	0.0	0.0	0.0	0.0	0.0	0.0	0.0	0.0	0.0	0.0
Yemen 1997	4.4	5.1	3.1	3.5	1.7	3.1	4.3	6.5	4.8	2.9	3.1
**Central Asia**											
Kyrgyz Republic 2012	0.1	0.0	0.0	0.2	0.4	0.0	0.0	0.0	0.0	0.0	0.0
Tajikistan 2012	0.1	0.3	0.3	0.0	0.1	0.1	0.4	0.0	2.5	0.0	0.1
Turkmenistan 2000	0.2	17.0	0.0	0.0	0.2	0.2	0.0	0.0	0.0	0.0	0.2
Uzbekistan 1996	0.4	2.0	0.0	0.3	0.0	0.0	0.0	1.9	0.0	0.0	0.4
**South and Southeast Asia**											
Bangladesh 2011	0.4	0.6	0.5	0.2	0.0	0.4	1.9	0.0	0.7	0.6	0.3
Cambodia 2010	0.0	0.0	0.0	0.0	0.0	0.0	0.0	0.0	0.0	0.0	0.0
India 2005-06	0.5	1.1	0.5	0.2	0.1	0.5	0.9	1.5	0.9	0.3	0.1
Indonesia 2012	0.2	2.4	1.3	0.1	0.2	0.1	0.9	1.4	1.3	0.5	0.1
Nepal 2011	2.9	9.5	2.4	1.3	0.6	2.0	6.5	12.0	4.5	2.7	1.0
Pakistan 2012-13	0.2	0.4	0.1	0.1	0.0	0.2	0.2	0.2	0.3	0.0	0.0
Philippines 2013	0.1	0.4	0.2	0.1	0.0	0.1	0.0	0.4	1.4	0.4	0.0
Sri Lanka 1987	0.2	NA	NA	NA	0.0	0.1	0.9	0.7	0.7	0.3	0.2
Thailand 1987	1.0	NA	NA	NA	0.2	1.0	1.8	4.2	0.3	1.0	1.2
Timor-Leste 2009-10	3.0	5.4	3.5	2.2	1.1	2.2	2.6	5.4	4.9	3.2	1.3
Vietnam 2002	0.1	0.3	0.1	0.0	0.0	0.0	0.6	2.0	1.1	0.0	0.0
**Latin America and the Caribbean**											
Bolivia 2008	0.9	2.9	1.4	0.5	0.2	0.7	1.3	2.2	3.3	1.2	0.2
Brazil 1996	0.6	1.7	1.2	0.3	0.2	0.3	1.6	1.9	0.3	1.0	0.3
Colombia 2010	0.2	1.1	0.8	0.1	0.0	0.2	0.4	1.1	1.0	0.5	0.1
Dominican Republic 2013	0.1	3.2	0.0	0.1	0.0	0.1	0.4	0.5	1.0	0.0	0.1
Ecuador 1987	2.2	NA	NA	NA	0.8	1.6	2.5	4.9	9.5	2.0	0.2
El Salvador 1985	7.6	NA	NA	NA	2.9	5.6	9.8	16.1	17.4	4.8	0.0
Guatemala 1998-99	1.2	2.7	3.2	0.4	0.3	0.7	1.2	2.9	2.3	1.0	0.0
Guyana 2009	0.6	0.0	0.0	0.7	0.0	0.8	1.1	0.6	0.0	0.2	0.7
Haiti 2012	1.9	2.7	2.1	1.7	0.5	2.1	3.3	2.9	3.6	1.7	1.1
Honduras 2011-12	0.6	3.3	1.0	0.5	0.1	0.7	1.2	1.8	3.2	0.8	0.1
Mexico 1987	2.6	NA	NA	NA	0.6	0.8	3.8	7.3	8.6	2.2	0.3
Nicaragua 2001	1.1	3.0	1.5	0.6	0.0	0.4	1.6	4.3	3.5	0.6	0.2
Paraguay 1990	0.5	2.7	0.2	0.4	0.0	0.1	1.4	1.2	0.0	0.7	0.0
Peru 2012	0.3	2.1	0.0	0.3	0.0	0.2	0.9	1.7	0.5	1.0	0.1
Trinidad and Tobago 1987	0.1	NA	NA	NA	0.0	0.0	0.5	0.0	0.0	0.2	0.0

a Data were restricted to the most recent live birth in the 3 years preceding each survey.

## DISCUSSION

These analyses have shown that far too many women—roughly 2.2 million based on recent data from 59 countries—delivered alone, with no one present. This practice is taking place predominantly in parts of the world with the worst maternal and newborn health indicators such as West and Central Africa and parts of East Africa. Complications during pregnancy and childbirth are a leading cause of death and disability among women in developing countries. Women who deliver alone are particularly vulnerable, as they do not even have access to the marginal support *any* attendance at birth confers. While we are not calling for anything less than for all mothers and newborns to have access to quality skilled care, ensuring that no mother delivers alone is an urgent moral and human rights imperative.

Giving birth alone takes place predominantly in West and Central Africa and parts of East Africa.

Recent research publications examined the issue of Nigerian women who gave birth with NOP.^6^^–^^8^ They found that the highest risk factors were poverty, rural residence, and rising maternal age. Our analysis confirms these findings both within Nigeria and across all countries studied. Nigeria, in particular, had severe wealth disparities in terms of women delivering alone (26% of the poorest women deliver alone compared with 2% of the wealthiest women). This finding suggests that the severity of inequity linked to delivering alone in Nigeria is exceptionally high in contrast to other countries included in this study. We also found a high proportion of urban women in Nigeria who gave birth alone (6%) in addition to a high proportion in rural areas (17%). This may be emblematic of the fact that Nigeria, as Matthews *et al*. have documented, is a country with large urban inequalities and a substantial urban rich advantage.^14^ In rural areas of Nigeria, the exceptionally large proportion of women who gave birth alone may be a direct result of a fewer number of facilities in rural areas.^15^ In terms of maternal age, there was a clear pattern across the countries included in our analysis that prevalence of giving birth alone increased with increasing maternal age. Given the constellation of extant risks for advanced maternal age, older mothers who deliver alone are particularly vulnerable to complications.^16^ However, in Nigeria and Niger, there was also a high proportion of young mothers who delivered alone; these young, often nulliparous, adolescent mothers who deliver alone are at significantly higher risk of developing obstetric fistula.^17^

Our analysis suggests that the drivers of delivery with NOP are of a structural nature, and not presumptively cultural. Across all 80 countries studies, the pattern was strikingly consistent, showing that women who gave birth alone were poor, had little education, and lived in rural areas. As the global community works to reduce inequalities in socioeconomic and health indicators, it will also likely have impact on eradicating delivery with NOP.

At the same time, evidence from Sokoto State, in northern Nigeria, suggests that the prevalence of delivery with NOP can be eliminated almost entirely through targeted actions by key stakeholders, along with education and advocacy, even in a population where poverty is pervasive, resources are scarce, and women are poorly educated. In 2008, the DHS reported that the prevalence of delivery with NOP in Sokoto State was 25%; by 2013, the prevalence had dropped to less than 1%.^18^^,^^19^

Programmatic experience in northern Nigeria suggests that the practice of giving birth alone can be reduced markedly through community education and advocacy.

Sokoto State is situated in the northwest corner of Nigeria, with an estimated population of just over 4.6 million in 2013, 80% of whom live in poverty.^20^^,^^21^ Data show that use of maternal health services in health facility settings is very low and has not improved in recent years; in both 2008 and 2013, 95% of married women in Sokoto State reported having delivered their most recent child at home.^18^^,^^19^ Furthermore, more than 80% of women in the state in 2013 reported that they had not accessed any antenatal care during their most recent pregnancy.^18^^,^^19^ The low uptake of maternal health services may be a direct result of few services available. There have been investments in increasing access to and the availability of health services in Sokoto State, resulting in a 26% increase in the number of government-run health facilities between 2009 and 2015, from 600 facilities to 756 facilities.^22^^,^^23^ Although efforts to increase the number of facilities have yielded results, the majority of women in Sokoto State, as noted above, still give birth at home.

The decline in delivery with NOP in Sokoto State coincided with multilevel discussions between government and civil society beginning in 2012 after the problem was first reported by JSI Research and Training Institute, Inc. (JSI) researchers.^7^ JSI researchers, working in Sokoto State with the Targeted State High Impact Project (TSHIP) funded by the United States Agency for International Development (USAID), shared their findings with government officials and civil society leaders. Whereas government officials in Sokoto State were surprised by the magnitude of the problem, community leaders were not. All parties agreed the status quo was not acceptable.

In addition to ongoing efforts to improve access to and use of quality maternal and newborn health services across Sokoto State, JSI/TSHIP began working with state-level leaders of Jama’atu Nasril Islam (JNI), Nigeria’s largest, most-networked, Muslim nonprofit aid group, to address the issue of women delivering alone. JNI leadership took charge of sounding an alarm and raising awareness among government and civil society leaders in Sokoto State. JNI also issued a call to eliminate the practice of delivery with NOP. Throughout all 244 wards in Sokoto State, JNI mobilized its local leaders and briefed them on the dangers associated with giving birth with NOP. Additionally, JNI called for local leaders to publicly discourage giving birth alone in homes. In 2012, JNI leaders trained Muslim clerics throughout the state to use appropriate verses from the Koran and *hadith* (collections of sayings or traditions of the Prophet Muhammad) to highlight the dangers of giving birth with NOP in relation to maternal and newborn mortality. Muslim clerics, with support from JNI, started to preach in favor of delivery with skilled assistance during Friday congregational prayers, wedding *fatihas* (religious ceremonies), and naming ceremonies. These efforts were successful in educating communities in Sokoto State on the dangers associated with giving birth alone, and ultimately changing the societal norms that had, in the past, condoned and facilitated the practice of delivering with NOP.

As a complement to these efforts, JSI/TSHIP also worked with the state government officials and communities to launch a 2,440-strong female community-based health volunteer (CBHV) team in 2012. These 2,440 CBHVs, representing 10 CBHVs per ward, were trained to counsel mothers on delivery with skilled attendance; the CBHVs made a total of 389,000 documented household visits in 2013 alone.^23^^,^^24^

The case of Sokoto State suggests that a process of community education and awareness has the potential to create ideation around norms in the short to medium term and can realistically accelerate the replacement of NOP-type deliveries with some type of attendance. This encouraging development is worth the attention of policy and civil society members across countries with relatively higher prevalence of deliveries with NOP. In itself, it is a call to action for leaders to act now alongside the broader implementation of SDG-related initiatives. The eradication of the practice of giving birth with NOP is only one step in ensuring that no mother or newborn dies of a preventable death. Ultimately, until every woman has safe, affordable, acceptable, and available maternal health services, there will remain barriers to achieving SDG targets 3.1 and 3.2.

### Study Limitations

Several limitations to our analysis should be noted. First, the DHS methodology collects information on women giving birth alone only through self-report.

Second, as the DHS is conducted in many countries, with multiple language groups, it is possible that women may not have understood the question correctly and thus that they incorrectly reported that their most recent birth occurred with “no one” present, despite efforts undertaken by the DHS to mitigate complications that may arise due to translation. The protocol for the standard DHS questionnaire not only asked women directly about who assisted with their most recent delivery but also probed those respondents who said “no one” assisted to determine whether any adults were present at the time of delivery.^25^

Another issue that must be noted, particularly in the Sokoto State context where the practice of giving birth with NOP has been so publicly and widely discouraged, is that DHS respondents may be reluctant to share that they have delivered alone. If women, due to social pressure and/or stigma associated with giving birth alone, are unwilling to report giving birth alone, it would be impossible to detect that, given the DHS methodology. Research on whether or not covert delivery with NOP occurs would be best informed through other study designs.

In Table 2 and Table 3, we were unable to include the number of women who gave birth. Unfortunately, the STATcompiler database neither provided specific sample sizes nor numerators and denominators for the percentages presented.

Also, many of the countries have not had a recent DHS survey, precluding determination of current *global* burden of delivering with NOP.

Furthermore, in our analyses of the burden of women who gave birth alone, there are some disagreements between the age parameters from the US Census Bureau (population of women aged 15–49) and the general fertility rate, as calculated by the DHS, which includes only women between the age of 15–44. According to the DHS, “The General Fertility Rate (GFR) is for the three years preceding the survey expressed per 1,000 women age 15–44. Note however that births to all women 15–49 are included in the numerator. In practice, there are very few births to women age 45–49 so the difference compared to restricting to births to women age 15–44 would be very small.”^26^

Finally, data available via STATcompiler could only be analyzed by using broad categories. Further research should use country-specific data to further explore the interactions between these predictive variables. Despite these data limitations, this analysis has shown that too many women are giving birth alone, and that grassroots advocacy and programmatic efforts are able to reduce the phenomena considerably, including in contexts where other socioeconomic determinants of health remain unchanged.

## CONCLUSION

Giving birth alone is a problem of important magnitude in many low- and middle-income countries, particularly in those countries with the worst maternal and newborn health indicators such as in West and Central Africa and parts of East Africa. Community education and awareness has the potential to change cultural norms in the short and medium term, accelerating the replacement of deliveries with no one present with some type of attendance. Ensuring that no mother delivers alone is an urgent moral and human rights imperative to prevent avoidable maternal and newborn deaths.

## Notes

**Peer Reviewed**

**First Published Online:** 2016 Mar 9
